# Lived Experiences of African Migrants Crossing the Strait of Gibraltar to Europe: A Cross-Cultural Approach to Healthcare from a Qualitative Methodology

**DOI:** 10.3390/ijerph18179379

**Published:** 2021-09-06

**Authors:** José Antonio Ponce-Blandón, Rocío Romero-Castillo, Nerea Jiménez-Picón, Juan Carlos Palomo-Lara, Aurora Castro-Méndez, Manuel Pabón-Carrasco

**Affiliations:** 1 Red Cross Nursing University Center, University of Seville, 41009 Seville, Spain; nejipi@cruzroja.es (N.J.-P.); jucapa@cruzroja.es (J.C.P.-L.); mpabon@cruzroja.es (M.P.-C.); 2 Faculty of Nursing, Physiotherapy and Podiatry, University of Seville, 41009 Seville, Spain; auroracastro@us.es

**Keywords:** migrant, vulnerable, public health, transcultural care

## Abstract

Background: The migratory flow from the African continent to Europe is intense and the European countries should apply a humanitarian, health and social response to this emerging problem. Migrants coming from Africa to Europe are a very vulnerable population. Healthcare professionals should be prepared for answering their needs from a transcultural approach, which requires a better understanding of this phenomenon. Thus, the aim of this study was to improve nursing and healthcare professionals’ awareness and better understanding of migrant life experiences during the migration journey. An exploratory descriptive qualitative research was conducted. In-depth interviews were conducted involving four key informants and content analysis were performed with the transcriptions. Results: Three themes merged: life situations in their countries of origin; motivations that led them to undertake the migratory journey; and experiences they lived during the migratory journey. The results described the dramatic experience and motivations for crossing the strait of Gibraltar from Africa to Europe, including feelings, fears, hopes and lived experiences. The determination of immigrants to fight for a better life opportunity and the physical damage and psychological consequences they suffer were revealed. Conclusions: This study would help healthcare professionals to better understand this complex reality and deliver culturally adapted care. Knowledge of the starting reality of these populations can help health professionals to incorporate a cross-cultural approach that improves the relational, ethical and affective competences to provide quality care to the migrant population, as well as the development of health measures to fight against inequalities suffered by these population groups.

## 1. Introduction

The migrating flows from the African continent into Europe are not a new phenomenon. Across the Strait of Gibraltar (the stretch of water between Africa and Spain), people desperately flee from poverty and try to seek well-being and to improve their quality of life in European countries. From January to September 2018, in Spain, almost 34,000 migrants arrived via the Mediterranean Sea, and 44,000 arrived via Italy and Greece [1]. Despite the fact that these movements are not new, the societies of the receiving countries are not prepared to understand the reasons for these flows and they do not always respond with solidarity. Prejudicial attitudes towards African migrants are based on perceived realistic and symbolic threats. Realistic threats are those that are considered as a menace to the host community, as well as threatening negative impacts which might affect general welfare. Symbolic threats relate to differences with the values and cultural identity of the host community [2]. Research has described rejection by a portion of European societies, which expressed fear that the migrant population may contribute to an increase of crime and insecurity, consume public resources, take advantage of services unsustainably or access work opportunities that are sometimes difficult to attain by the local population [3]. According to Pereira, Vala and Costa–Lopes [4], discriminatory behaviors and opposition to immigration are consequences of preconceived attitudes justified by threat perceptions.

Healthcare is one of the basic and essential needs of migrants at arrival and in the subsequent stages [5]. The vulnerability of undocumented migrants regarding their health status, living conditions and barriers to access to healthcare and social welfare is recognized. The impact of living conditions on the quality of life and psychological distress of the migrant population has been described. Leaving their home country because of war or persecution, hunger, having experienced abuse, economic challenges, homelessness and higher age are factors associated with a poorer quality of life and distress [6,7]. From the physical health approach, the review conducted by Woodward, Howard and Wolffers [8] pointed out that digestive, skin, musculoskeletal, respiratory, dental and gynaecological issues, in chronic and acute forms, are the most common health problems among migrant adult and child patients.

Despite their vulnerability and poor health status, and the fact that healthcare is universally and equably accessible in the European countries, there is evidence of inequalities between migrants and non-migrants in access to healthcare services [9]. Biswas, Kristiansen, Krasnik and Norredam [10] identified limited medical rights, arbitrariness in nurses and healthcare professionals’ attitudes, fear of being reported to the police, poor language skills, lack of a network with local citizens, lack of knowledge about the healthcare system and lack of knowledge about informal networks of healthcare professionals as barriers to the healthcare system. Other barriers identified were the complexity of their health problems, limited socio-cultural skills among providers, linguistic issues during consultations and added administrative efforts [8]. These obstacles led to alternative health resources that may have negative consequences on the migrants’ health, such as self-medication, advice needing to be sought from doctors in their country of origin or borrowed health insurance cards [10] as well as an overloading of emergency services due to an inadequate usage of non-emergency services [11].

Nurses are the front-line health professionals who would be dealing with their demands in the emergency services, hospital settings or in the primary care centers [12,13]. However, authors agree that they may not be prepared to provide an effective response from a transcultural perspective [14]. Although nurses are willing to make no distinctions in patient care because of their migratory status, they have reported some challenges that need to be addressed: language barriers, issues of false identification, insecurities about the correct standard procedures and not always being able to provide appropriate care [10]. As De Vito, De Waure, Specchia, Parente, Azzolini, Frisicale, Favale, Teleman and Ricciardi identified [15], fear of stigma, cultural and language barriers and differences in religious practices and customs represent an obstacle to accessing healthcare and to providing the appropriate care that migrants need. Healthcare professionals who are culturally competent and are sensitive to cultural differences are needed to lower the cultural and language barriers [16]. Training in transcultural care and better understanding the experience suffered by this vulnerable population should be included in the actual nursing curricula in Europe, as societies are becoming more culturally plural and heterogeneous, swarming with minority groups of migrants [17].

The healthcare professionals’ response to the immigration challenge should focus on equality, with concrete actions according to the social determinants of health, such as the risk of exclusion this population is exposed to. It is necessary to better understand the causes of these migrating movements and the reality of their precarious subsistence in our countries, in addition to the discrimination they suffer. It is also essential for current and future healthcare professionals to be better prepared in terms of interpersonal and communication skills as well as transcultural competence that would allow for offering a more effective and holistic care [17,18].

The purpose of this paper is to improve nurses and healthcare professionals’ awareness of the life experience of the migrant population from Africa into Europe, including motivations and their backgrounds.

## 2. Materials and Methods

Exploratory descriptive qualitative research was conducted to gain insight into the lived experience of undocumented migrants during their journey across the Strait of Gibraltar.

### 2.1. Hypothesis

1. The motivations for the migrants’ journey are related to a hostile departure situation.
2. The experience lived during the migratory journey has profound consequences for the individual on a physical and emotional level.
3. The expectations of migrants before the trip are the improvement of their quality of life and better development opportunities.

### 2.2. Setting, Sample and Procedure

The study was conducted in a specialized Red Cross welcome center. It is a multifunctional health and social support center that provides emergency assistance, health care and support to migrants to continue their migration process. During 2018, 7698 migrants who arrived on the coast irregularly were attended to in this center [19]. Basic healthcare was provided according to their needs, as well as information, support and guidance through the mediation process. The center had the capacity to attend to 600 people simultaneously and a large team of psychologists, social workers, cultural mediators, translators, nurses, doctors and Red Cross volunteers.

Individual interviews were conducted during the initial consultation, with a nurse in charge with the purpose of gathering data about their life situations in their countries of origin, the motivations to undertake this journey and the experiences they lived through. Purposive sampling was used to recruit the key informants (KI). The established inclusion criteria were: (i) migrant attended the Red Cross basic health service; (ii) older than 15 years; and (iii) not having any health conditions that require hospital referral. The established exclusion criteria were: (i) staying in the center for less than 48 h (due to a lack of time to conduct in-depth interviews in this emergency context), (ii) not being a fluent French-speaker (needed in order to have an in-depth interview and provide informed consent). From August to September 2018, 458 migrants were attended to by the basic healthcare service in this center, but only 24 of them met the inclusion criteria. Most of them stayed at the Red Cross basic health center for less than 48 h, so the research team considered they were not there for long enough to build a trusting relationship required for an interview of this nature. Due to their vulnerability and the harshness of the experience, feelings of fear and mistrust emerged; therefore, only four KI agreed to participate. Despite the reduced number of interviews, data saturation was reached. They responded to the four main profiles identified to diversify and enrich the discourse: (i) young man traveling on his own; (ii) woman traveling on her own; (iii) unaccompanied minor; and (iv) woman accompanied by her family. 

### 2.3. Data Generation

To obtain data, in-depth interviews were conducted following the assisted autobiographies interview technique described by Ruiz–Olabuénaga [20]. A minimum of two interviews were carried out for each of the KIs, with an approximate duration of 30 min each, on two different days. The interviews were carried out by the researcher accompanied by a cultural mediator who translated the questions and answers into the participants’ language (French), and who had received relevant practical training in the interview protocol. The interviews were guided by three open-ended questions which aimed to obtain the following information: (i) the life situation in their countries of origin; (ii) motivations to undertake the migratory journey; and (iii) the experiences they had lived through during the migratory journey. All participants’ responses were recorded, transcribed and subsequently shown to the KI for approval, in order to perform the content analysis. 

### 2.4. Data Aalysis

The Hermeneutic phenomenology approach was used to understand the meaning that participants assigned to their experiences [21]. For data analysis, content analysis for transcriptions was used from a narrative perspective [20]. Two researchers independently coded the data collected in the interviews and inductively compared their interpretations, identifying relevant quotations, units of meaning, themes and sub-themes. The reliability and rigor of the qualitative information recorded was carried out through a triangulation process between both researchers, analyzing the information separately before reaching an agreement about results. 

### 2.5. Ethical Considerations

Interviews were carried out with the assistance of a cultural mediator. The language spoken was French, which was at least the second language of all the participants, thus guaranteeing understanding of the language. They took place in a private room with only three people attending: the participant, researcher and cultural mediator. Disruptions were avoided. All possible doubts that might have arisen during the interviews were clarified, facilitating the interruption of the interview and even the abandonment of the interview in case the interviewees felt distressed. Re-telling their experiences evoked sadness and sorrow, so psychological support was provided.

Participants had prior knowledge of their right to leave the interviews at any time and voluntarily agreed to participate in the study by providing informed consent. To prevent the identification of the participants, proper names and specific locations of the stories (not the countries of origin) were deliberately changed, using “nicknames”. Data were recorded anonymously and treated with extreme confidentiality.

Participants’ safety was guaranteed. Interviews were conducted at least 24 h after arrival, in order to give them time to settle down and calm their emotions. Their basic needs were met, and good physical and psychological conditions were checked prior interviews. A healthcare team was available during the interviews, in case of need. 

The study was approved by the Research Ethics Committee of Red Cross University Nursing College (CUECR 08/2018).

## 3. Results

Following the criteria of variability established to diversify and enrich the discourse, four interviews were conducted. The participants’ characteristics are summarized in Table 1. The ages ranged from 16 to 36, while 50% were men and 50% were women. They came from Sub-Saharan and northern Africa countries, and traveled on their own (*n* = 3) or with their family (*n* = 1).

### 3.1. Life Situation in Their Countries of Origin

All participants lived in difficult situations before starting the trip, with tough economic, family and emotional conditions. They usually came from large families without economic or social resources, including KI-3. He had six brothers and sisters, his mother sold dates in the local market from 6 in the morning until sunset, and his father could not work due to a pulmonary disease and needed to be cared for. Most of them went to school for the first period, even though access to the school was not easy to obtain. As KI-1 reported, he walked to school 10 km every day until he was 14.

*“I enjoyed school very much. I liked learning and playing with other kids. But I felt I had to help my family, I had to do something for them.”*

(KI-1).

When they reach adolescence, the school cannot offer them a longer education, additional training or job opportunities, as all of these options are scarce. They must find a way of living, but in most cases, they just wander around in the streets with friends of the same age and conditions, playing soccer in improvised fields or stealing some fruit or anything to sell so they can eat something, as KI-3 reported. Girls have other options, which are sometimes equally dramatic: to leave poverty, KI-2′s father arranged a marriage with a 52-year old rich man, who already had two wives and lived in a nearby town.

*“I didn’t want to marry that man; I didn’t want to be just someone’s wife. I wanted to get a job and rule my own life.”*

(KI-2).

The armed conflicts present in some countries of origin aggravate these problems. That occurred for KI-4, who lived in a small settlement mainly inhabited by the Wolof ethnic group, in an area in constant conflict due to the actions of the Democratic Forces Movement (DFM) opposing the Senegalese government. She married a miner who worked in the phosphate operations of the area. They had five children. They barely made a living with her husband’s low salary but, despite that, they were a united family. The social context became more and more challenging due to the continuous clashes among the different ethnic groups and the DFM’s terrorist actions. Her older son left at age 19 to earn a living and her second daughter, then 15-years old, ended up in the hands of a pimp who used her to provide sexual services to tourists. Her 13-years old twin sons were shot dead on a day that they went to cut wood in the forest, amidst a terrorist act by the DFM guerrilla in which they murdered another 14 youngsters and children.

### 3.2. Motivations to Undertake the Migratory Journey

Motivations to undertake the risky migratory journey identified during the interviews were remarkably similar. They wanted to escape from a tough reality and fight for an opportunity (KI-2). This motivating goal could be extended to their relatives; for example, KI-1 also wanted to take his family out of poverty and KI-4 wanted to provide a brighter future to her 9-years old little girl. Sometimes, they are strongly motivated by previous successful experiences of their acquaintances.

*“Home wasn’t a safe place anymore. We wanted to give our little girl the opportunity that her brothers and sister didn’t have.”*

(KI-4)

KI-3 mentioned that his friend crossed the Strait a few months ago and that he had managed to settle in Sabadell with some sporadic jobs.

*“I thought that if I did the same and join my friend in Spain, maybe I would go to the Camp Nou and watch Leo Messi playing.”*

(KI-3)

### 3.3. Lived Experiences during the Migratory Journey

It is an extremely difficult, dangerous and long journey. KI-1 mentioned that it took him more than 3 years. He walked more than 3000 km from Guinea to Algeria, accompanied by a friend who also wanted to go to Europe searching for a better future. He lived with his friend in the streets across 5 different countries for 3 years, trying to enter Morocco. He made a living as best he could, sleeping in the streets, selling fruits and rural products and even managed to save some money. Eventually, they managed to shake off the Moroccan Police and made it to the Melilla fence, determined to cross it at any cost. With his friend, they managed to climb by the concertinas, but the Spanish Civil Guard discovered them. He managed to let himself loose from the concertina, fell into the void and tore the sacro-lumbar zone of his back along the way; but that did not stop him from running to the Moroccan mounts, although his friend remained trapped among the wires.

*“I was so determined in getting through that I didn’t realize I got hurt. I could only think in running as fast as I could, and not looking backwards.”*

(KI-1).

He managed to get a place in a small boat that set sail, sailing for more than 13 h crammed with another 68 people on board. Fortunately, all of them reached Spain safely. 

Migration is also awfully expensive, considering their low or non-existent incomes. KI-4 were told that they had to invest all that they had. They sold everything they possessed and managed to get to Morocco, where they survived for 4 months begging and sleeping in the streets. Unfortunately, they were robbed of all the money they carried with them. After collecting the money again, they managed to get a place in a zodiac to cross the Strait, setting sail with another 35 people on board. The 14 long hours journey was not hard after so much previous suffering.

*“We were very excited and hopeful, a brand-new future was waiting for us on arrival. Despite the horrible experience, I felt happy.”*

(KI-4).

In some cases, although the journey is expensive, it also comes with a huge emotional costs. According to her story, KI-2 paid all the money her mother could raise to fund a journey to France. She ended up in a people trafficker hands and for more than 14 months, she wandered with another group of women through different parts of Mali, Mauritania and Morocco. During the migratory journey, she was raped by the people trafficker and by many of his contacts, as a requirement to pay off the contracted “debt”. She was even humiliated and battered by the Police and finally made it to the coast, where she shared a precarious boat with another 60 people.

*“I lost my dignity, I didn’t feel like a human being, I was just an object they could use. Sometimes I felt like that was not happening to me, but someone else, like I was an observer from outside.”*

(KI-2).

Two men of the boat forced her and other women to sit with their legs around the engine’s gasoline tank, ending in a drain of sea water mixed with gasoline. When they managed to get close to the coast of Tarifa (Spain), enough to be intercepted by a Spanish sea rescue boat she barely felt her legs. 

The migration journey across the Strait of Gibraltar is often managed by mobs. KI-3 mentioned that he was contacted by someone who offered him a place in a boat to cross the Strait. He and his 4 friends persuaded their families to sell some properties and to jointly pay the 4000 euros that the capo had asked for from them. It was going to be a quick and safe operation without any deportation risks because they were underage.

*“The man of the boat told us that there won’t be any problems because of my age. Everything would be OK as I was under 18 years and I trusted him.”*

(KI-3).

They finally got on board of what seemed to be a small fishing boat. When they were in the middle of the sea, without knowing where they were, the boss abandoned the five of them offshore in an inflatable boat. His clothes got all soaked and the cold started to doze him off until he was awakened by the shout of one of his friends when he saw the light of a ship. A Spanish sea rescue ship had located them and they were safe now.

*“I was very scared, I was terrified. I was very cold and I thought that I was going to die there in the sea, that I would not come out of that darkness.”*

(KI-3).

## 4. Discussion

This qualitative study described the life experiences of the migrants during their journey from Africa to Europe through a phenomenological approach. The results would contribute to a better understanding of their vulnerable situation, in order to improve the quality of the healthcare provided. 

Life experiences described in this study may help to enhance empathy and diversity awareness among the receiving population. As Murray and Marx identified [2], the receiving countries show prejudicial attitudes, perceived realistic threats, and intergroup anxiety, especially towards unauthorized migrants. Regarding health, discriminatory attitudes are reflected in difficulties in accessing healthcare for migrants. From a legal point of view, the United Nations and the Council of Europe recognize a right to health for undocumented migrants, so governments should guarantee the availability, accessibility, acceptability and quality of health services, especially for specific groups such as women and children [22]. Although most of the European countries are able to rely on universal health insurance systems, they only provide emergency services for undocumented migrants [23] or certain services under specific conditions (e.g., infectious diseases) or subgroups (e.g., pregnant women, children) [15]. According to Suess, Ruiz–Pérez, Ruiz–Azarola, and March–Cerdà [24] legal restrictions of access to the health system could be due to the impact of the current economic crisis and the lack of knowledge of the migrants’ perspectives. Other factors that prevent equitable legal access to the health systems have been identified, such as previously enacted restrictive policies, cost concerns, poor influence of healthcare professionals on policy development and concerns about the side effects of generous treatment of undocumented migrants on other groups [25]. From participants’ approach, including both healthcare professionals and migrants, further formal and informal barriers in the access to healthcare by migrants have been described, involving cultural and language barriers and a lack of knowledge and awareness [15]. 

However, the migrant population is a vulnerable group in terms of health and is susceptible to complex health problems, with socio-economic roots and implications for the entire community. The risk of being excluded from the healthcare assistance is higher when living with HIV [26]. Results from a qualitative study conducted by Arrey, Bilsen, Lacor and Deschepper [27] revealed that women living with HIV in Belgium perceived discriminatory and stigmatizing attitudes in health care settings, shown as delayed or denied care, excessive precautions, blame and humiliation. Fear of stigma and discrimination is also a barrier to HIV and tuberculosis testing among migrant populations, which hinders sustainable preventive measures [28,29]. Perceived discrimination and stressful life events, like the journey experiences described by the participants in the present study, are associated with cardiovascular risks among migrant populations [30]. As Myhrvold and Småstuen found [7], the level of psychological distress is extremely high among undocumented migrants and this is linked to intense feelings such as exploitation, loneliness, powerlessness, fear and a sensation of being constantly worried. Socio-economic conditions also influence the mental health of migrants. Their insecure living and working conditions were found to be associated with increased stress, depressive, anxiety, sleeping and somatic symptoms [8]. According to Forte, Trobia, Gualtieri, Lamis, Cardamone, Giallonardo, Fiorillo, Girardi and Pompili [31], migrant populations are exposed to a higher risk of suicide attempts due to language barriers, worrying about families back home and separation from family. In summary, the evidence shows that migrant populations have physical and psychological health needs related to their life experience that health systems in host countries should address.

The interviews included in this study revealed that the migrants crossing the Strait of Gibraltar risk their lives during the journey. Although this journey is noticeably short in distance (the closest point between the Morocco and Spain coasts is about 14 km wide), it could take more than 24 h [32]. Sadly, some migrants die during the journey and many arrive on the Spanish coasts with physical and psychological consequences. They are often victims of occasionally unpredictable climate conditions and, above all, of the manipulation of their lives by the mafia that operate in the Strait of Gibraltar [33]. Young people who cross the Strait pay the mafia around 4500 euros, which is equivalent to lifetime’s family savings. For them, this means the opportunity of a new and happier life, even if could entail permanent separation from their families. The meagre value the mafias which operate these migrating routes confer to the life of a human being sadly implies for the migrants a risk of death. For the mafia this value is a very profitable business [1]. For the European societies, it represents another cause of fear, rejection and distrust that healthcare providers should be aware of [15,34].

The harshness of the testimonies collected in this study highlights the need for healthcare for migrants upon arrival on the Spanish coast. An adequate coverage for migrants is needed to address newly arrived migrants’ health problems: communicable diseases, such as respiratory, gastrointestinal and dermatologic infections; non-communicable diseases, including chronic conditions, mental and social problems [35]. Given the high prevalence of infectious diseases in African migrants, accurate screening and tailored protocols are recommended, as they could lead to severe health problems for the individual in the case of delayed or non-existent treatment, representing a high cost to the public health system and possible transmission in the receiving country [36]. 

Described experiences from this research highlight the particularly difficult experience for female migrants. Their conditions in their country of origin are usually less favorable, as they are more vulnerable to discrimination from the receiving countries and are exposed to health problems and sexual exploitation [37]. Female migrants face a double discrimination due to being a woman and being a migrant, and they are at risk of experiencing sexual abuse, rape and violence. Therefore, reproductive health needs are often neglected [38]. The review conducted by Balaam, Akerjordet, Lyberg, Kaiser, Schoening, Fredriksen, Ensel, Gouni and Severinsson [39] pointed out that migrant women are particularly vulnerable when pregnant and giving birth in European countries. They have problems finding meaning in their new country to be able to cope, communicate, connect and achieve a safe pregnancy and childbirth [39]. According to Fair, Raben, Watson, Vivilaki, Van den Muijsenbergh, Soltani and the ORAMMA team [40] pregnant migrant women not only struggle with the communication and language barrier, but they also find it difficult to go through the health system to establish caring relationships and meet their needs as they go beyond pregnancy, including to assist with psychosocial-emotional and economic issues. Sami, Quack Lötscher, Eperon, Gonik, Martinez de Tejada, Epiney and Schmidt [41] suggested some measures to alleviate negative migrant women’s experiences during pregnancy and childbirth, including providing information in multiple languages, accessible interpreters, trained nurses to guide migrants through the health system and a cultural competence-training program for healthcare providers. Therefore, our results are in line with previous similar studies that claim the need to develop an emergency gender policy for irregular migrant women, especially when providing healthcare in police-controlled settings [33,42]. 

All the lived experiences narrated in the interviews collected in this study may contribute to improving the quality of healthcare provided during the phases of rescue and emergency care. Transcultural competency is particularly necessary for nurses, as a range of barriers to healthcare for undocumented migrants has been reported by experts, including a shortage of culturally sensitive care and the complexity of the social needs of migrants. In fact, attempts at improving healthcare for migrants should consider communication skills, which are necessary to provide culturally sensitive care and to ensure enough resources for this [43]. 

The American Nurses Association emphasized that the interaction between a health care provider and a client involves three cultural systems: that of the client; the health care provider; and the organization [44]. These interactions may cause conflict and barriers to care, thereby causing more disparities. Therefore, cultural competence must be fostered in organizational settings [45].

Procedure and culture affect the quality of care provided and can increase or ameliorate disparities in care and outcomes. Some of the conditions of an organization are: a high value placed on diversity; the capacity to evaluate the dynamics inherent in cultural interaction; and flexibility in adapting care with an understanding of cultural diversity. The absence of these conditions can lead to a deep distrust of fellow citizens and can aggravate or intensify health disparities and poor outcomes [46].

The Code for Nurses emphasizes that the need for health care is universal, regardless of all individual differences. The nurses are duty-bound to implement nursing services without prejudice and with the utmost respect for human needs and for the client as a person [47]. 

### Limitations

The number of in-depth interviews could be considered as limited. This was due to the distinctively vulnerable circumstances of the participants, as well as the short stay of the immigrants in the center where the study was conducted. Despite the small number of participants and the expected variability of life experiences, the themes of the qualitative analysis emerged easily and were quickly identified, reaching the saturation of information with the profiles of selected participants. The procedure for recruiting and selecting the participants was similar to in other qualitative studies of a comparable nature [33,42,48], despite the differences in the number of participants.

The nature of the situation of participants could be considered as a limitation. The researchers avoided causing KI to relive the process during their narration, as that could create anguish in them. This may have reduced the quantity of the information provided. It is possible that the immigrants had health problems that the nurses did not detect with the naked eye in these interviews. All the measures of protection of psychological integrity of the participants described in the methodology were considered. Another limitation that should be considered is information lost in translation. Interviews were conducted in French, which was the participants’ second language, and the transcriptions were translated into Spanish for analysis. Some meanings and connotations may not have reached the research team, or the information may have been reduced.

## 5. Conclusions

This paper aims to contribute to a better understanding of the life experiences of migrants. Their testimony would help increase awareness around what is behind the migrating flow phenomenon from Africa into Europe. Knowing the social and personal realities that motivate the migrating population could be relevant to complement the care provided, and for the promotion, prevention and rehabilitation of migrants’ health from a transcultural perspective. Culturally competent health care will lead to greater patient satisfaction, improved clinical outcomes and greater cost efficiency.

Healthcare professionals should have a broader training in transcultural competences in order to offer more effective care to the migrating population, based on a closer comprehension of their lived experiences. Madeleine Leininger, the first individual to define “cross-cultural nursing”, explained that it is a formal area of study and work focused on care and based on the culture, beliefs of health or illness, values and practices of people, to help them maintain or regain their health, or cope with their disabilities or death. Cross-cultural competence was essential when we completed interviews and assessments of the patients. It was also important when we made health education and sanitary recommendations. This is due to the fact that sometimes health workers make the mistake of assessing and recommending health care options to patients following our own culture and customs. For example, if we want a patient to improve his diet, we must know what diet he is eating and know his culture. This will make dietary recommendations more successful.

It is necessary to include a transcultural approach in the training of European nurses which would improve the interpersonal, ethical and affective competences in order to provide quality care. It would also facilitate the development of health-relevant interventions against inequalities and to address the vulnerability issue that the immigrant population presents in our context. Despite the information available on the morbimortality of the immigrant population, it is necessary to continue research studies which offer a vision of irregular immigration from a humanitarian, social and health perspective.

## Figures and Tables

**Table 1:** Socio-demographic data of participants.

Participant	Sex	Age	Travel Status	Country
KI-1	Male	19	Alone	Guinea Konakry
KI-2	Female	20	Alone	Ivory Coast
KI-3	Male	16	Alone	Morocco
KI-4	Female	36	Husband and son	Senegal

## Data Availability

Data is not available due to a requirement of the ethics committee. For ethical issues and patient privacy and following a respect for interviews and statements.
